# Comparison of telemedicine-assisted psychotherapy, exercise therapy, or a combination of both in patients with post-COVID-19 syndrome (TelPoCo): study protocol for a randomized controlled trial

**DOI:** 10.1186/s13063-025-08968-7

**Published:** 2025-07-20

**Authors:** Sebastian Beyer, Mariel Nöhre, Isabell Pink, Sebastian Häckl, Nele Henrike Thomas, Frank Klawonn, Uwe Tegtbur, Martina de Zwaan, Sven Haufe

**Affiliations:** 1 Department of Rehabilitation and Sports Medicine, Hannover Medical School, Carl-Neuberg-Str. 1, Hannover, 30625 Germany; 2 Department of Psychosomatic Medicine and Psychotherapy, Hannover Medical School, Hannover, Germany; 3 Department of Respiratory Medicine and Infectious Diseases, Hannover Medical School, Hannover, Germany; 4 Institute for Biostatistics, Hannover Medical School, Hannover, Germany; 5 Helmholtz Center for Infection Research, Biostatistics, Brunswick, Germany

**Keywords:** Post-COVID-19 syndrome, Exercise therapy, Psychotherapy, Multimodal therapy

## Abstract

**Background:**

Post-COVID-19 syndrome (PCS) presents in a multitude of ways, with fatigue, physical constraints, and diminished quality of life being common symptoms. It is becoming increasingly clear that unimodal behavioral interventions do benefit all PCS patients. Adherence to and response to isolated psychotherapy or physical activity interventions vary greatly, with certain patients benefit more from one form of therapy, or even a combination, than others do. The study aims to compare the effects of a single exercise therapy, psychotherapy, and a combination of both therapies.

**Methods:**

The study will be conducted as a prospective, randomized controlled, open-label trial with 3 treatment arms (exercise therapy, psychotherapy, and combined therapy). According to the sample size calculation, 65 participants will be enrolled in each group. The primary outcome is the change of PCS fatigue symptoms from baseline to 3 months, estimated by the Fatigue Assessment Scale. Secondary endpoints include changes in further measures of fatigue (Chalder Fatigue Skala, Multidimensional Fatigue Inventory, Post-exertional Malaise Scale, Bell Scale), health-related quality of life (Short Form-36 and Brief Illness Perception Questionnaire), anxiety and depression (Hospital Anxiety and Depression Scale), and work ability (Work Ability Index). The intervention lasts for 3 months and includes online therapy sessions of 50 min every 2 weeks or in case of lack of concentration or fatigue this could be split to two 25-min sessions (all equating to a total of 300 min of specialist care). The psychotherapy adopts a short-term and coping-oriented approach based on the unique requirements of each patient from a psychotherapeutic perspective. Exercise therapy involves a personalized physical activity plan customized to suit the patient’s requirements, with tracking day-to-day physical activity along with daily moderate endurance and strengthening workouts. An ANCOVA model, including the stratification factors sex and BMI, will be used for the primary analysis of Fatigue Assessment Scale. Significance tests will be based on the group differences in least square means and corresponding 95% CIs.

**Discussion:**

Due to the current relevance of the issue, the unclear evidence so far, and the lack of appropriately powered randomized studies, it is crucial to assess potentially effective concepts for treating patients with PCS. Future therapy decisions will benefit from answering the question of whether combined therapies hold a significant advantage over unimodal therapeutic approaches, as well as identifying predictors that indicate an advantage of certain therapies for particular patients.

**Trial registration:**

ClinicalTrials.gov NCT06042751. Registered on 21 September 2023.

**Supplementary Information:**

The online version contains supplementary material available at 10.1186/s13063-025-08968-7.

## Administrative information

**Table:** 

Title {1}	Comparison of telemedicine-assisted psychotherapy, exercise therapy, and a combination of both in patients with post-COVID-19 syndrome (TelPoCo): study protocol for a randomized controlled trial
Trial registration {2a and 2b}	The study has been registered on 21 st September 2023 at ClinicalTrials.gov (ClinicalTrials.gov Identifier: NCT06042751)
Protocol version {3}	Protocol version 3.1, protocol date 2024/12/06
Funding {4}	The study is funded by the COVID-19 Research Network Lower Saxony (COFONI), with funding from the Ministry of Science and Culture of Lower Saxony, Germany (14–76,403-184) under the project number 9LZF23
Author details {5a}	Sebastian Beyer^1^*, Mariel Nöhre^2^*, Isabell Pink^3^, Sebastian Häckl^4^, Nele Henrike Thomas^4^, Frank Klawonn^5^, Uwe Tegtbur^1^, Martina de Zwaan^2^*, Sven Haufe^1^* *These authors contributed equally ^1^Department of Rehabilitation and Sports Medicine, Hannover Medical School, Hannover, Germany ^2^Department of Psychosomatic Medicine and Psychotherapy, Hannover Medical School, Germany ^3^Department of Respiratory Medicine, Hannover Medical School, Germany ^4^Institute for Biostatistics, Hannover Medical School, Germany ^5^Helmholtz Center for Infection Research, Biostatistics, Germany
Name and contact information for the trial sponsor {5b}	Hannover Medical School Department of Rehabilitation and Sports Medicine Carl-Neuberg-Str. 1, 30,625 Hannover Phone: + 49–511–532 5499; fax: + 49–511–532 8199 Email: sportmedizin@mh-hannover.de
Role of sponsor {5c}	Central data collection and verification of reportable events; notifying investigators and reporting of SUSARs, AEs, and SAEs within required timelines; submission of the annual progress reports, including safety summary and deviations; preparing and publishing study data in publicly accessible journals The funder of this study had no role in the study design and will have no role in data collection, data analysis, data interpretation, writing of the report, or decision to submit for publication

## Introduction

### Background and rationale {6a}

A post-COVID-19 syndrome (PCS) as defined by the World Health Organization (WHO) “occurs in individuals with a history of probable or confirmed SARS-CoV-2 infection, usually 3 months from the onset of COVID-19 with symptoms that last for at least 2 months and cannot be explained by an alternative diagnosis” [1]. Estimates indicate that PCS occurs in up to 10% of all infected persons, affecting not only severely ill patients but also those with a mild or even asymptomatic course [1–3]. The courses of PCS are varied, but all lead to limitations in everyday life due to chronic fatigue, exercise intolerance, dyspnea, neurocognitive impairments, muscle pain, sleep disorders, and/or headaches [4].

To date, few randomized controlled trials (RCT) have been published on the efficacy of physical rehabilitation on fatigue in PCS patients. A current meta-analysis lists three controlled studies with one of them showing a between-group improvement, with a pooled significant decrease in fatigue severity [5]. More recent RCTs either showed differences in fatigue between exercise and usual care groups after 4 to 12 weeks of intervention [6, 7] or no significant effects, despite some indices of improved physical performance [8, 9].

Research on the effectiveness of psychotherapeutic approaches in patients with PCS-related fatigue is still limited, with some trials currently ongoing. A pilot study by Huth et al. shows potential to improve fatigue as well as other psychosocial outcomes in patients with PCS [10]. In 2023, a study presenting a psychotherapeutic intervention to treat fatigue in patients with sarcoidosis was published. The online mindfulness-based cognitive behavioral therapy approach was able to significantly improve fatigue, anxiety, depression, and overall health status in patients with sarcoidosis-associated fatigue [11].

Taken together, current data suggests that psychotherapeutic and exercise therapy concepts are beneficial in some but not all conducted RCTs. Nevertheless, a combination of both therapy approaches should have a greater effect on fatigue than unimodal therapies, a research question not addressed so far. In this context, a comprehensive care model should be home-based and supported by personal coaching via telemedicine, which allows reaching as many patients as possible at a low threshold [7, 12–14]. Therefore, the hypothesis of this randomized three-arm study is that 3 months of individual, telemedicine-supported therapy, which combines psychotherapy and exercise contents, improves fatigue more than psychotherapy or exercise therapy alone. An additional aim of the study is to investigate predictors for the responsiveness of the three conducted therapy approaches.

### Objectives {7}

The primary objective of the study is to show that 3 months of individual, combined psychotherapy and exercise therapy improve fatigue more than psychotherapy or exercise therapy alone.

Another aim is to investigate predictors for the responsiveness of psychotherapy or exercise therapy or the combination of both.

### Trial design {8}

This is a randomized, controlled, three-arm, open-label, monocentric intervention trial to demonstrate superiority of a combined therapy for PCS fatigue symptoms over monotherapeutic approaches. PCS patients will be randomized in a 1:1:1 ratio stratified by sex (male/female) and body mass index (BMI) (< 30/≥ 30 kg/m^2^) to the 3 treatment groups.

## Methods: participants, interventions, and outcomes

### Study setting {9}

The baseline assessment takes place at Hannover Medical School, the university hospital in Hannover, Lower Saxony, Germany, which is the only study side. The study intervention will be performed telemedically by the therapists based at Hannover Medical School.

### Eligibility criteria {10}

This study will include participants who are 18 years of age or older, have been diagnosed with COVID-19 disease by real-time quantitative reverse transcriptase polymerase chain reaction (PCR) or antibody testing, and have experienced persistent fatigue for more than 3 months with a Fatigue Assessment Scale (FAS) score of ≥ 22. Participants currently enrolled in another interventional study, those with clinically relevant acute or chronic infections or organ damage caused by COVID-19, or those with suicidal tendencies or severe mental illness (such as mania or the acute phase of schizophrenia) requiring acute treatment are not eligible for this study. Participants who are unable to participate in the physical training intervention due to severe illnesses or functional limitations, as determined by the examining study doctor, will also be ineligible to participate.

### Who will take informed consent? {26a}

Informed consent is obtained by a trained and delegated physician (study investigator). The physician informs the patient about the objective, content, period, possible risk and harms, and data protection regulation of the study. Patients meeting the eligibility criteria receive both an oral explanation of the study and a participant information sheet with a participant consent form (see supplementary material 1 and 2 for original language and english language, respectively). Written informed consent is obtained prior to inclusion by discussing the nature, objectives, possible adverse events, and all other unresolved issue associated with the participation in the study. A copy of the information sheet and the signed and dated consent form are supplied to the patient providing written consent.

### Additional consent provisions for collection and use of participant data and biological specimens {26b}

N/A: only data available in the electronic medical record of the patients are collected for the purposes of this trial. No additional consent provisions are needed.

## Interventions

### Explanation for the choice of comparators {6b}

PCS is a complex condition in which pathophysiological processes have not been understood yet. At the same time PCS presents itself in many different symptoms and effects on daily life. This situation calls for holistic therapeutic approaches that target many different symptoms. Based on the rational that a combination of therapeutic interventions (psychotherapy and exercise therapy) addresses different effect mechanisms, a synergistic effect and an improved outcome are expected from the combination therapy. To be able to show this synergistic effect, monotherapeutic psychotherapy and exercise therapy were chosen as comparators.

### Intervention description {11a}

The study consists of three arms, each including 300 min of online therapy sessions over a period of 3 months, and a follow-up period of 3 months (6 months after BSL). The therapy consists of 300-min online intervention with 50-min sessions every 2 weeks (alternatively up to 12 × 25 min every week). The interventions are led by an exercise scientist and a psychological psychotherapist, respectively.

In the exercise therapy intervention, an individual exercise plan will be developed during the first online session based on the results of the aptitude test and the participant’s descriptions and routines. This exercise plan includes managing daily activities, a heart rate limit, moderate endurance and strength exercises, as well as stretching and relaxation exercises. To account for the physical inactivity caused by fatigue in many participants, light strengthening exercises (e.g., chair squats and wall push-ups) and shorter endurance exercises (e.g., walking, ergometer training, Nordic walking, cycling) will be usually performed at the beginning. If participants are currently receiving therapy, such as regular physiotherapy or ergotherapy, this will be integrated into the exercise plan and planned around it. The exercise plan aims to increase the frequency of strength and endurance training stimuli and to adjust the intensity to individual circumstances without causing overload or crashes. The activities are recorded using a wearable device. To ensure subjectivity of the intensities, the patients will keep a training diary during the intervention, in which the subjective feeling of exertion is assessed using the Borg scale and the daily feeling in the morning and in the evening (scale 1–10) is documented. During therapy sessions, activities will be discussed and exercise plans and the time between sessions will be considered. This includes wearable data, training diaries, sleep patterns, recovery, and existing therapies such as physiotherapy and occupational therapy. Other strenuous activities, such as cognitive activities, will also be taken into consideration as well as the physiology of training, fatigue, and recovery. The exercise therapy aims to improve reduced resilience and fatigue without overwhelming patients with volume or intensity. This will be achieved through a comprehensive approach that allows patients to engage in continuous, daily physical activity.

In the psychotherapeutic intervention, a modularized, coping-oriented short-term therapy based on the individual needs of the patient will be performed taking into account the specific deficits identified in the psychosomatic evaluation at baseline. The sessions focus on the following topics: (1) psychoeducation, (2) mindfulness regarding one’s own stress limits, (3) time and energy management based on self-observation to prevent crashes, (4) improving self-care and resources, (5) differentiate between helpful and less helpful coping strategies, and (6) consideration and treatment of mental comorbidity.

Within the sessions, starting points will be identified with the patients, which the patients can work on independently between sessions.

In the combined intervention, 12 sessions à 25 min will be performed alternating between psychotherapy and exercise therapy. The contents of the interventions are the same as described; however, due to the same amount of therapy in all three study groups, the treatment content in the combined study group is conveyed in a condensed form.

### Criteria for discontinuing or modifying allocated interventions {11b}

One or more of the following circumstances may lead to premature termination of the study for an individual subject: withdrawal of the subject’s consent, occurrence of an exclusion criterion, therapy discontinuation by the patient (not attending therapy visits or documenting discussed therapy contents), and other circumstances that would to lead to worsening of PCS symptoms if continuing the study. For the benefit and interest of the participant, the investigator can terminate study participation at any time if serious side effects or other unforeseeable circumstances occur that endanger the health of the participant. In this case, the Ethics Committee of Hannover Medical School will be informed.

### Strategies to improve adherence to interventions {11c}

Prior to the commencement of the study, the entire research team underwent comprehensive training, which encompassed the principles of good clinical practice, the specifics of the study protocol, and the respective responsibilities of each team member.

A significant portion of the study is conducted online or via telemedicine, as patients with post-COVID-19 syndrome can exhibit considerable limitations in their resilience and mobility. As part of the study, only one on-site appointment is required. All other study assessments and treatments are completed online. This primarily includes the therapy appointments and the questionnaires used to assess the study outcomes. To facilitate patient convenience, the 50-min therapy sessions may be divided into two 25-min sessions. Patient reported outcome measures are also completed online, and respondents may interrupt and resume the questionnaires at their convenience. Additionally, the Physiotools app offers access to video demonstrations of therapeutic exercises from the field of exercise therapy, which can be viewed at any time.

### Relevant concomitant care permitted or prohibited during the trial {11d}

When a participant receives concomitant care that does not impair the ability to participate at the online-guided intervention, the study can be continued as planned. However, a simultaneous participation in a different trial focusing on the improvement of fatigue or quality of life is not possible. Additionally, participants are asked not to begin new treatments, unless they are urgently required for medical reasons. Changes in the therapy during the trial were documented.

### Provisions for post-trial care {30}

Post-trial care is not planned for this trial. If needed, patients will be referred to regular care that is available for post-COVID syndrome, i.e., physiotherapy, ergotherapy, or psychotherapy. The sponsor (Hannover Medical School) is providing an insurance, even without fault, to cover its liability as the requesting party in case of harm caused to the patient by participation in the study.

### Outcomes {12}

#### Primary outcome

This study examines the change in fatigue, as measured by the total score on the FAS [15], following a 3-month intervention period. The FAS is a 10-item assessment scale comprising 5 questions pertaining to physical fatigue and five questions (items 3, 6–9) pertaining to mental fatigue. Total scores range from 10 to 50. A total FAS score of less than 22 indicates the absence of fatigue, a score of 22 or above indicates the presence of fatigue, and a score of 35 or above indicates the presence of extreme fatigue.

#### Secondary outcomes

1. Health-related quality of life was assessed using the Short Form-36 Health Survey (SF-36) [16]. The 36 items comprising the SF-36 are designed to reflect eight domains of health, including physical functioning, physical role, pain, general health, vitality, social functioning, emotional role, and mental health. The range is 0–100, with higher scores indicating a superior quality of life. Furthermore, a physical and mental composite score can be calculated.
2. Depression and anxiety, as measured by the Hospital Anxiety and Depression Scale (HADS-D) [17]: The HADS-D questionnaire comprises 14 items pertaining to the two subscales for anxiety and depression. Subscale scores range from 0 to 21, with higher scores indicating more severe anxiety or depression. Values can be interpreted as normal (0–7 points), mild (8–10 points), moderate (11–14 points), or severe (15–21 points).
3. The degree of physical and psychological fatigue severity, as measured by the Chalder Fatigue Scale (CFS) [18]. The 11-part self-report instrument comprises a total scale and two subscales, namely physical and psychological fatigue. The maximum total score is 33, with higher scores indicating an increased level of fatigue. The maximum score for physical fatigue is 21, derived from seven items, while the maximum score for mental fatigue is 12, derived from four items. Furthermore, a binary code can be calculated for each of the 11 items (0 and 1 = 0; 2 and 3 = 1) in order to identify cases of severe fatigue. A total score of four or above is indicative of severe fatigue.
4. Post-exertional malaise, as measured by the Post-Exertional Malaise Scale (PEM-Scale) [19]. The PEM-Scale employs five distinct 5-point Likert scales to quantify the frequency or severity of PEM symptoms and a 7-point Likert scale to assess the duration of PEM. This results in a maximum score of 46.
5. Multidimensional fatigue, as measured by the Multidimensional Fatigue Inventory questionnaire (MFI-20) [20, 21]. The MFI-20 is a 20-item self-assessment instrument comprising five subscales: (1) general fatigue, (2) physical fatigue, (3) decreased activity, (4) decreased motivation, and (5) mental fatigue. Each subscale contains four items, with respondents indicating their level of agreement on a 5-point Likert scale (1 = strongly agree, 5 = strongly disagree). Higher scores indicate higher levels of fatigue.
6. Disability, as measured by the Bell Disability Scale [22]. The Bell Disability Scale comprises 11 statements pertaining to the level of physical function. The scale is rated on a 10-point scale, with 0 indicating severe disability and 100 indicating full health.
7. Work ability, as measured by the Work Ability Index (WAI) [23]. The WAI is a self-report questionnaire comprising seven items pertaining to work, work ability, and health. The total score, which ranges from 7 to 49, is interpreted as an indicator of work ability, with higher scores reflecting greater work ability.
8. Illness perception, as measured by the Brief Illness Perception Questionnaire (IPQ) [24]. The IPQ is a tool designed to assess perceptions of cognitive and emotional illness. The abbreviated IPQ comprises 8 novel items and a portion of the causal scale that was previously employed in the IPQ-R. All items except the causal question are rated on a scale of 0 to 10. Five of the items assess cognitive representations of illness, namely consequences (item 1), time frame (item 2), personal control (item 3), treatment control (item 4), and identity (item 5). Two assess emotional representations: concern (item 6) and feelings (item 8). One item evaluates the comprehensibility of illness (item 7). An open-response item (item 9) requests patients to list the three most important causal factors for their illness.

### Participant timeline {13}

See Table 1.

**Table 1:**
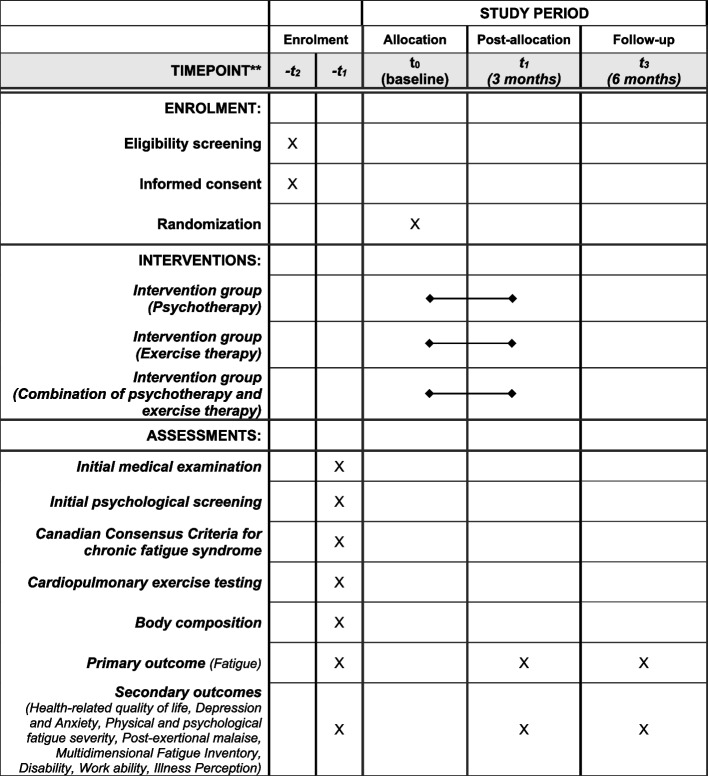
Schedule of enrollment, interventions, and trial assessments

### Sample size {14}

The sample size calculation is performed for the primary endpoint change from baseline to 3 months in the PCS fatigue symptoms estimated by the FAS. There is currently no published data available regarding the effect of a 3-month single psychotherapy or physical interventions compared to a combined approach on PCS fatigue-specific symptoms. Thus, the assumptions for the sample size calculation are based on preliminary data from a self-conducted pilot study (exercise therapy) and a trial by Kahlmann et al. (psychotherapy), in which a 3-month mindfulness-based, behavioral therapy in patients with chronic lung disease was investigated [8, 11]. A FAS change from baseline at 3 months of 2.0 (standard deviation (SD) = 5.0) for the physical therapy and 4.5 (SD = 5.8) for the psychotherapy, respectively, is assumed. Due to the fundamentally different therapy approaches, it is assumed that there will be more responders and less drop-outs in the combined therapy group compared to each monotherapeutic approach. Thus, a slightly additive effect of 7.0 with a SD of 5.0 is anticipated for the combination therapy. The sample size calculation is based on the comparison between the psychotherapy and the combined therapy approach since the difference of the FAS change for this comparison is assumed to be smaller than between the exercise and combination therapy. With a two-sided type-I-error of 5%, a power of 80%, a pooled SD of 5.0, and a difference of 2.5 in the FAS-change between psychotherapy and the combination therapy using a *t*-test for two independent samples, a sample size of *n* = 64 patients per group is required. With a Satterthwaite test assuming unequal variances (SD = 5.8 for the psychotherapy, SD = 5.0 for the combination therapy), a super additive effect of 7.2 would be necessary to achieve a power of 79.9% with the same sample size. Despite the co-primary group comparison approach, the power does not need to be increased to 90% because of the distinctly larger effect difference between the exercise therapy and the combination therapy (5.0). For a sample size of *n* = 64 patients per group, the power for this comparison of the single exercise therapy with the combination therapy would be > 99%.

To achieve a power of 80%, a total sample size of *n* = 195 patients (65 patients per group) is needed to demonstrate superiority of the combination therapy over monotherapeutic approaches. It is assumed that using an ANCOVA model adjusted for the stratification factors for the primary analysis will only increase the power compared to applying a *t*-test. All sample size calculations were performed using nQuery 9.2.1.0.

### Recruitment {15}

To recruit participants, we will conduct information events and distribute information material at Hannover Medical School, particularly in our outpatient clinics, as well as place newspaper articles and online advertisements in the Hannover region. In addition, the main Volkswagen factory in Wolfsburg (Lower Saxony, Germany) will contribute to recruitment of participants. Therefore, we will organize a series of online and print advertisements at the factory premises with support of the occupational health centers and the personnel department, which will also distribute advertisements via email and the company intranet to employees.

## Assignment of interventions: allocation

### Sequence generation {16a}

Permuted block randomization with variable block length stratified by sex (male/female) and BMI (< 30/≥ 30 kg/m^2^) is used to allocate patients in a ratio 1:1:1 to the therapy arms. The randomization code is generated in SAS 9.4 using the RANTBL function as default random-number generator. The seed for the randomization procedure is randomly chosen. The randomization code including seed and algorithm is electronically stored in a separate folder and access is granted only for responsible persons involved in generating the randomization list.

### Concealment mechanism {16b}

The randomization is performed centrally by the Institute for Biostatistics; therapy allocation is conducted via fax randomization—the randomization request can either be sent via email or fax. Clinicians involved in the study only receive the result of the allocation via email after the randomization process has been completed. During the course of the trial, clinicians neither have access to the randomization list nor are they directly or indirectly involved in the randomization process.

### Implementation {16c}

The allocation sequence was generated in SAS 9.4 by an independent statistician within the Institute for Biostatistics. Patients can be enrolled by authorized clinicians who are also allowed to request randomization of the patient. The randomization team on duty within the Institute for Biostatistics is responsible for assigning a therapy to each randomized patient according to a trial-specific standard operation procedure. The allocation of the patient is documented on the randomization form which is signed by the randomization team and sent via fax or email to the pre-determined authorized person who requested the randomization. The randomization process is in line with good clinical practice (GCP) guidelines.

## Assignment of interventions: blinding

### Who will be blinded {17a}

Study physicians, exercise scientist, psychotherapist, outcome assessors, and study participants are not blinded for the allocated intervention.

### Procedure for unblinding if needed {17b}

N/A, due to the nature of the study the interventions are not blinded.

## Data collection and management

### Plans for assessment and collection of outcomes {18a}

The data obtained from the screening are recorded manually and subsequently transferred to an electronic case report form (eCRF). The data are subjected to periodic verification for accuracy through random sampling and continuous monitoring. The primary outcome is completed by the patients themselves as part of the initial examination and then verified through a double-entry process before being transferred to the eCRF.

All other study outcomes are collected via online questionnaires at baseline, at the end of treatment after 3 months, and at follow-up, 3 months after the end of treatment. This system precludes the possibility of missing data, as patients are only permitted to proceed or complete the process if all requisite entries have been made. In the event that the questionnaires are not received within a week, the patients are contacted on a weekly basis via telephone or email to remind them of the necessity to complete the questionnaires. The data obtained from the online questionnaires can be exported and evaluated using an evaluation file. The evaluation file was created and verified in advance. Furthermore, the entire process is subject to continuous monitoring.

### Plans to promote participant retention and complete follow-up {18b}

All efforts are made to keep patients in the trial and to collect complete data, in particular for the primary endpoint, even after a premature therapy and/or trial discontinuation. In case that the questionnaires are not completed, the patients are contacted again via telephone or email to remind them to take part in the follow-up.

### Data management {19}

The primary data collection is performed via two web-based systems. The self-reported variables will be captured in the SoSci-Survey Tool, a web-based system provided by the Hannover Medical School (MHH) for creating online questionnaires, sending participation invitations, and downloading data (e.g., as a CSV file). The responsibility for the data collection and sending of participation invitations in SoSci-Survey lies within the Department of Rehabilitation and Sports (MHH) as well as the safe and correct transfer of the data from SoSci-Survey to the Institute for Biostatistics. A standardized process for validated data transfer is provided by the Institute for Biostatistics. All further data (e.g., demographics, safety data) are collected and filed via the web-based EDC-system secuTrial®. secuTrial® is a browser-based GCP-conform EDC-system by the company interactive system for capturing, storing, and managing patient data in clinical trials, non-interventional trials, and patient registers. The eCRF of the trial is based on a Data Dictionary created collectively by the Institute for Biostatistics, the Department of Rehabilitation and Sports Medicine, and the Department of Psychosomatic Medicine and Psychotherapy of the MHH. The MHH provides the server for secuTrial®. The Institute for Biostatistics is responsible for joining the data from SoSci-Survey and secuTrial®, data export, data storing, as well as off-site monitoring. All available data will be exported as a SAS file from secuTrial® and monitored every quartile. Moreover, all available data from SoSci-Survey will be exported as a csv file and provided every quartile to the Institute for Biostatistics. Responsibility for data quality of the raw data as well as for an adequate digitalization of the data lies within the Department of Rehabilitation and Sports Medicine and the Department of Psychosomatic Medicine and Psychotherapy of the MHH. The Institute for Biostatistics is responsible for calculation and derivation of variables, e.g., scores from questionnaires. For quality assurance, all partners of the trial can be audited by responsible employees of the Institute for Biostatistics. Further information on data management and data quality is detained in a data management agreement in the currently active version.

### Confidentiality {27}

All study data collected will be treated confidentially in accordance with the requirements of the data protection law of the federal state Lower Saxony (Germany) and European Privacy legislation. All investigators and trial staff must comply with these requirements in relation to the processing of personal data, with regard to the collection, storage, processing, and disclosure of personal information. Personal data is not accessible to third parties. All data collected will be evaluated in a pseudonymized form, which means that a code number replaces identification features such as name, date of birth, and address so that an assignment to the participant is only possible via an additional reference list. If the study results are published, the confidentiality of personal data will be maintained. According to German law, recorded medical data will be deleted after the usual retention period has expired (currently 10 years after publication).

The online-based implementation of the intervention is supported by online applications. The online video conversations take place via Medflex (medflex GmbH, Konstanz, Germany), a system for video consultations provided by Hannover Medical School. Medflex will only be used as software for the conversation between participants and investigators; no sensitive data, health data, or confidential documents will be shared. The data recorded with the wearable activity tracker (Garmin Forerunner 55, Olathe, USA) are uploaded to the database of our partner Fitrockr (Fitrockr (c/o Digital Rebels GmbH), Berlin, Germany) via an internet connection to a server located in Germany and provided from there to the study staff. The identification of the study participant will be pseudonymized as a code number. The above-mentioned companies have an existing cooperation agreement with Hannover Medical School and have been assessed for data protection and data security within this framework.

### Plans for collection, laboratory evaluation, and storage of biological specimens for genetic or molecular analysis in this trial/future use {33}

N/A, such evaluations are not planned.

## Statistical methods

### Statistical methods for primary and secondary outcomes {20a}

Primary goal of the trial is to show superiority of the combination therapy (exercise therapy and psychotherapy) regarding fatigue symptoms assessed by the FAS in PCS patients compared to the respective monotherapeutic approach. The trial is considered successful if the FAS score is significantly improved for the combination therapy compared to the single exercise therapy and psychotherapy (two-sided alpha of 0.05). Therefore, the trials aims to show superiority of the combination therapy versus the exercise therapy as well as the psychotherapy. Both comparisons are tested co-primarily with a two-sided significance level of 0.05, i.e., both comparisons must be statistically significant for trial success. The primary estimand of the trial is the FAS score and is characterized as follows:

- Intervention: psychotherapy, exercise therapy, and combination therapy (psychotherapy and exercise therapy).
- Population: patients ≥ 18 years with ongoing symptoms (longer than 3 months) after proven COVID-19 infection (PCR or antibody test) and a FAS score ≥ 22.
- Variable: absolute change from baseline to 3 months (end of intervention).
- Population-level summary: mean difference in the absolute change from baseline in the FAS score.
- Intercurrent events: patients who die due to an intercurrent event or prematurely end the therapy (because of an adverse event, insufficient efficacy, or other reason) are included in the primary analysis irrespective of this experience, i.e., intercurrent events are handled by applying the treatment policy strategy. Missing values will be replaced conservatively (i.e., in favor of the null hypothesis) using the last-observation-carried-forward (LOCF) approach.

The null and alternative hypotheses for the primary estimand for the co-primary comparisons of the combined therapy versus the respective monotherapeutic approach are defined as follows with µ = FAS (3 months) minus FAS (baseline):

- H_10_: µ (FAS-change) Combi = µ (FAS-change) Psychotherapy
- H_11_: µ (FAS-change) Combi ≠ µ (FAS-change) Psychotherapy
- H_20_: µ (FAS-change) Combi = µ (FAS-change) Exercise therapy
- H_21_: µ (FAS-change) Combi ≠ µ (FAS-change) Exercise therapy

In line with the treatment policy strategy regarding intercurrent events, the primary analysis is performed in the intention-to-treat (ITT) population, i.e., patients are analyzed as randomized regardless of the actual received therapy or premature trial termination. The change from baseline to 3 months in the FAS score is analyzed using an analysis of covariance (ANCOVA) model with the change from baseline to 3 months in the FAS score as dependent variable, and therapy group, FAS baseline value, and the stratification factors sex (male/female) and BMI (< 30/≥ 30 kg/m^2^) as independent variables. Superiority of the combination therapy compared to the respective monotherapeutic approach is shown (H_10_ and H_20_ are rejected) if (A) improvement in the FAS scores (estimated by least square means) are higher in the combination therapy group than in both monotherapy groups and (B) the *p* value of the associated *F*-test for both comparisons is < 0.05.

Secondary endpoints are analyzed in line with the primary analysis.

### Interim analyses {21b}

No interim analysis is planned.

### Methods for additional analyses (e.g., subgroup analyses) {20b}

Further sensitivity analyses for the primary endpoint include the performance of a Satterthwaite test (Welch test) for the comparison of the FAS score between the three treatment arms in the case of unequal variances in the respective groups, and in case of falsely documented stratification factors (if revealed after randomization), the repetition of the primary analysis with the correct values. Subgroup analyses will be performed at least for the stratification factors of the randomization sex (male/female) and BMI (< 30/≥ 30 kg/m^2^).

Adverse (AE) and serious adverse events (SAE) are documented and descriptively evaluated. AEs and SAEs are compared between the therapy groups using a chi-square test.

### Methods in analysis to handle protocol non-adherence and any statistical methods to handle missing data {20c}

The primary analysis will be performed in the ITT population, which is in line with the treatment policy strategy to handle intercurrent events, i.e., patients will be analyzed as randomized regardless of the experience of an intercurrent event. Missing values will be replaced using a conservative LOCF approach. A per-protocol (PP) and a complete-case (CC) analysis will be conducted in line with the primary analysis as sensitivity analyses. Patients who (a) do not prematurely ended the trial (i.e., no withdrawal of informed consent, intolerable adverse events, occurrence of an exclusion criterion, premature therapy end by the patient, and other circumstances that would compromise a patient’s health in case of further trial participation) and (b) have a FAS score at 3 months (end of intervention, visit 2) are included in the PP population. The CC population comprises patients who are (a) included in the PP population, (b) participated in at least 5 out of 6 sessions á 50 min or 10 out of 12 sessions á 25 min, (c) have a FAS score at 3 months (end of intervention, visit 2), and (d) do not show any other compliance violations (e.g., not using the wearables, large time period between planned and performed visit (</> 10 days)).

### Plans to give access to the full protocol, participant-level data and statistical code {31c}

The study data (as SPSS or SAS data set) and the statistical code will be provided upon request by the Institute for Biostatistics at the study end and after finalization of the clinical study report. Before data bank closure, no data will be disclosed.

## Oversight and monitoring

### Composition of the coordinating center and trial steering committee {5d}

The Department of Rehabilitation and Sports Medicine acts as the coordinating center. The coordinating investigator and the principal investigator together with an expert psychiatrist, composing the trial steering committee, have bi-weekly meetings to discuss the study progress. The coordinating investigator is responsible for the day-to-day communication with the local principal investigators from the participating centers. Twice a year, members of the study group updating the funder of the study (COFONI network) on the study’s progress during scheduled meetings.

### Composition of the data monitoring committee, its role and reporting structure {21a}

There is no data monitoring committee for this trial, but as part of data monitoring the data management team at the Institute for Biostatistics will check the collected and documented data in the SoSci-Survey Tool and the eCRF for completeness and plausibility.

### Adverse event reporting and harms {22}

Adverse and serious adverse events have been predefined by the study team. They are both documented in the study registry (ClinicalTrials.gov Identifier: NCT06042751). Serious adverse events and other unintended effects are discussed in the investigator team and reported to the responsible ethic committee.

### Frequency and plans for auditing trial conduct {23}

The data management team will provide a monitoring report every month for the general status and missing data. In addition, once a month the randomization status will be send to study PIs and study coordinators.

### Plans for communicating important protocol amendments to relevant parties (e.g., trial participants, ethical committees) {25}

During the trial, all protocol amendments and revised informed consent form are sent to the central EC for their review. Amendments are not implemented without prior review and documented approval/favorable opinion from the EC. After approval, important protocol modifications are directly communicated to the relevant parties and included in all necessary study documents.

### Dissemination plans {31a}

It is planned to publish the results of the study in a scientific journal under open access conditions and to present them to the specialist public at scientific congresses. In addition, a summary of the results in layman’s terms is planned for the participants and other interested citizens. It is planned to make the study manual available to other interested practitioners.

## Discussion

Even though the exact number of people affected is unknown, it can be assumed that a relevant number of patients is affected by PCS. The pathomechanisms behind the development of PCS are not yet sufficiently well understood, so that no causal therapies are available. Information on treatment trials trying to improve symptoms and dealing with the disease and the associated functional impairment are also limited [10]. Both, psychotherapeutic and exercise therapy concepts have been suggested as beneficial interventions for PCS, yet results are still inconsistent and not conclusive. A combination of both therapy approaches might have a synergistic effect on fatigue compared to unimodal therapies, a research question not addressed so far. Therefore, we planned an interventional trial based on previous literature-based and own experiences from the treatment of patients with PCS [8, 25] to compare the combination therapy to psychotherapeutic and exercise therapy concepts alone.

The importance of telemedical treatment services has grown considerably in recent years. This method is also planned for the current study, as it is believed to be particularly suited to severely impaired patients with limited energy and severe fatigue [26]. However, there is still a paucity of data globally and from Germany on the acceptance and feasibility of such treatment programs for PCS patients [27]. It is essential to elucidate the extent to which these forms of medical intervention are accepted by the participants in our study, as this will inform about the feasibility and transferability of these treatment options into routine clinical practice. Possible problems in the course of the study will include whether patients will be sufficiently recruited and whether acceptance and willingness to participate in our study will be sufficiently high. In this context, the course and prevalence of PCS to date and in the near future is hardly to project which might affect the recruitment process and duration of the study.

## Trial status

Protocol version: 3.1, protocol date: 6 December 2023. Date of start recruitment: December 12, 2023. Approximate date of recruitment completion: March 2025.

## Supplementary Information

Supplementary Material 1.

Supplementary Material 2.

## Data Availability

Data will be uploaded to the data repository of the funder (COFONI network) and will be publicly available.

## Abbreviations

AE: Adverse event
BMI: Body mass index
CC: Complete-case
CFS: Chalder Fatigue Scale
eCRF: Electronic case report form
GCP: Good clinical practice
FAS: Fatigue Assessment Scale
HADS-D: Hospital Anxiety and Depression Scale
IPQ: Brief Illness Perception Questionnaire
ITT: Intention-to-treat
LOCF: Last-observation-carried-forward
MFI-20: Multidimensional Fatigue Inventory questionnaire
MHH: Hannover Medical School
PCR: Real-time quantitative reverse transcriptase polymerase chain reaction
PCS: Post-COVID-19 syndrome
PEM: Post-exertional malaise
PP: Per-protocol
RCT: Randomized controlled trials
SAE: Serious adverse events
SD: Standard deviation
SF-36: Short Form-36 Health Survey
WAI: Work Ability Index
WHO: World Health Organization
