# Supplementary material for: Comparison of telemedicine-assisted psychotherapy, exercise therapy, or a combination of both in patients with post-COVID-19 syndrome (TelPoCo): study protocol for a randomized controlled trial
Source: Trials. 2025 Jul 20;26:251. doi: 10.1186/s13063-025-08968-7 (PMC12278562; doi:10.1186/s13063-025-08968-7)
Supplement: Supplementary file 1 — Supplementary Material 1. [file 13063_2025_8968_MOESM1_ESM.pdf]

Medizinische Hochschule Hannover

Einwilligungserklärung

Randomisierter Vergleich von telemedizinisch gestützter Psycho- und Bewegungstherapie und deren Kombination bei Patient:innen mit Post-Covid19-Syndrom (TELPOCO)

....
Name des/der Probanden/in in Druckbuchstaben

geb. am ....

Ich bin in einem persönlichen Gespräch durch die aufklärende Ärztin/ den aufklärenden Arzt

……..
(Name der Ärztin / des Arztes)

ausführlich und verständlich über den Ablauf sowie über Wesen, Bedeutung, Risiken und Tragweite der klinischen Studie aufgeklärt worden. Ich habe darüber hinaus den Text der Patienteninformation und die Datenschutzerklärung, sowie die hier abgedruckte Einwilligungserklärung gelesen und verstanden. Ich hatte die Gelegenheit, mit dem Untersuchungsleiter über die Durchführung der klinischen Studie zu sprechen. Alle meine Fragen wurden zufrieden stellend beantwortet. Ich habe den Sinn und Zweck der Studie und alle Ablaufdetails verstanden.

Ich hatte ausreichend Zeit, mich zu entscheiden.

Mir ist bekannt, dass ich jederzeit und ohne Angabe von Gründen meine Einwilligung zur Teilnahme an den Untersuchungen zu jedem Zeitpunkt zurückziehen kann (mündlich oder schriftlich), ohne dass mir daraus Nachteile entstehen.

Ich bestätige mit meiner Unterschrift, dass ich mit den Untersuchungen sowie einer zukünftigen Kontaktierung zur langfristen Erforschung des Post-Covid19-Syndroms einverstanden bin. Meine Teilnahme erfolgt unvergütet.

Ich war innerhalb der letzten 14 Tage nicht akut krank und werde jede Veränderung des Allgemeinbefindens während der Untersuchung dem Untersuchungsteam sofort mitteilen.

Ich erkläre mich bereit, an der oben genannten Studie freiwillig teilzunehmen.

Ein Exemplar der Probanden-Information und -Einwilligung habe ich erhalten. Ein Exemplar verbleibt im Institut.

....
Datum Unterschrift des/r **Probanden/in**

Ich habe das Aufklärungsgespräch geführt und die Einwilligung des Probanden eingeholt.

....
Datum Unterschrift des aufklärenden **Untersuchers**

Datenschutzeinwilligung für die Nutzung eines Wearables sowie Handy-Applikationen zur Onlinebetreuung im Rahmen der Studie „Randomisierter Vergleich von telemedizinisch gestützter Psycho- und Bewegungstherapie und deren Kombination bei Patienten mit Post-Covid19-Syndrom (TELPOCO)“

……………………………………………………………………

Name des/der Patienten/in in Druckbuchstaben

Ihre vom Wearable aufgezeichneten Daten werden in pseudonymisierter Form (also in einer Form, bei der Identifikationsmerkmale wie Name und Anschrift durch ein Kennzeichen – z.B. eine Codenummer – ersetzt sind, so dass eine Zuordnung zu einer Person nur über weitere Hilfsmittel – etwa eine Referenzliste – und nur innerhalb der MHH möglich ist) auf einem Server außerhalb der MHH gemäß den datenschutzrechtlichen Vorschriften, insbesondere der DSGVO, gespeichert und verarbeitet. Mithilfe des Wearables und eines mobilen Endgerätes erfolgt die Synchronisierung über die Fitrockr Hub App. Die Fitrockr Hub App ist auf dem mobilen Endgerät zu installieren. Zur Auswertung der Trainingsdaten werden Ihre Gesundheits- und Aktivitätsdaten auf einem Server der Firma Fitrockr übertragen.

Zugang zu den pseudonymisierten Daten haben nur direkte Mitarbeiter:innen der Klinik für Rehabilitations- und Sportmedizin MHH. Die Zuordnung der pseudonymisierten Daten zu Ihrer Person erfolgt auch nur durch Mitarbeiter:innen der Klinik für Rehabilitations- und Sportmedizin.

Zu den aufgezeichneten und übermittelten Daten gehören Aktivitätsdaten wie Schritte und Bewegungsdauer einer Aktivität, als auch Gesundheitsdaten wie Ihre Herzfrequenz und die GPS-Daten Ihrer Bewegungen. Die GPS-Daten Ihrer Bewegung werden ausschließlich aufgezeichnet, wenn Sie manuell auf dem Wearable eine Sportaktivität aktivieren. Die Funktion der GPS-Aufzeichnung kann auch grundsätzlich deaktiviert werden.

Ich habe verstanden, dass die Daten, die ich im Rahmen der Trainingstherapie preisgeben werde, in pseudonymisierter Form gesammelt und ausgewertet werden. Das bedeutet in einer Form, bei der Identifikationsmerkmale wie z.B. Name, Geburtsdatum und Anschrift durch eine Codenummer ersetzt sind, so dass eine Zuordnung zu meiner Person nur über eine weitere Referenzliste möglich ist. Nur die Mitarbeiter der Klinik für Rehabilitations- und Sportmedizin können meine Identität nachvollziehen. Im Falle einer Veröffentlichung der Projektergebnisse bleibt die Vertraulichkeit persönlicher Daten gewahrt. Das Datenschutzgesetz des Landes Niedersachsen findet Anwendung.

Die Verwendung der Angaben über meine Gesundheits- und Aktivitätsdaten erfolgen nach gesetzlichen Bestimmungen und setzen, gemäß Artikel 6 Abs. 1 lit. a der DS-GVO, vor der Teilnahme an der klinischen Studie folgende freiwillig abgegebene Einwilligungserklärung voraus, das heißt ohne die Einwilligungserklärung kann keine Teilnahme an der Studie erfolgen.

Mir ist bekannt, dass diese Einwilligung jederzeit schriftlich oder mündlich ohne Angaben von Gründen widerrufen werden kann, ohne dass mir dadurch Nachteile entstehen. Die Rechtmäßigkeit der bis zum Widerruf erfolgten Datenverarbeitung wird davon nicht berührt. In diesem Fall kann ich entscheiden, ob die von mir erhobenen Daten gelöscht werden sollen oder weiterhin für die Zwecke der Studie anonymisiert verwendet werden dürfen.

Ich wurde darüber informiert und willige ein, dass meine Daten nach Beendigung oder Abbruch der Studie mindestens zehn Jahre gesetzeskonform aufbewahrt werden. Danach werden meine personenbezogenen Daten gelöscht, soweit nicht gesetzliche, satzungsmäßige oder vertragliche Aufbewahrungsfristen entgegenstehen.

Ich habe das Recht, Einsicht in die Daten zu nehmen, die während der Studie erhoben werden. Sollte ich dabei Fehler in den Daten feststellen, so habe ich das Recht, diese durch den Studienarzt korrigieren zu lassen. Ich habe das Recht über die betreffenden personenbezogenen Daten Auskunft zu erhalten (einschließlich unentgeltlicher Überlassung einer Kopie) sowie ggf. deren Berichtigung oder Löschung zu verlangen. Des Weiteren haben Sie das Recht auf Einschränkung der Verarbeitung Ihrer personenbezogenen Daten (Art. 18 DS-GVO), auf Datenübertragbarkeit (Art. 20 DS-GVO) und ein allgemeines Widerspruchsrecht (Art. 21 DS-GVO).

Ich weiß, dass meine Teilnahme an dieser Studie freiwillig ist und ich meine Einwilligung jederzeit ohne Angabe von Gründen widerrufen kann, ohne dass mir daraus eventuelle Nachteile entstehen.

Ich bin mit der Erfassung meiner persönlichen Daten einverstanden.

....	....
Datum	Unterschrift des/r **Patienten/in**
