# Supplementary material for: Comparison of telemedicine-assisted psychotherapy, exercise therapy, or a combination of both in patients with post-COVID-19 syndrome (TelPoCo): study protocol for a randomized controlled trial
Source: Trials. 2025 Jul 20;26:251. doi: 10.1186/s13063-025-08968-7 (PMC12278562; doi:10.1186/s13063-025-08968-7)
Supplement: Supplementary file 2 — Supplementary Material 2. [file 13063_2025_8968_MOESM2_ESM.pdf]

Medizinische Hochschule Hannover

Declaration of consent

Randomised comparison of telemedicine-supported psychotherapy and exercise therapy and their combination in patients with post-Covid19 syndrome (TELPOCO)

....
Name of the interested patient

Date of birth ....

I have been informed in a personal interview by the informing physician

.... (Name of the study physician)

I have been informed in detail and comprehensibly about the procedure as well as the nature, significance, risks and scope of the clinical trial. Furthermore, I have read and understood the text of the patient information and the data protection declaration, as well as the declaration of consent printed here. I had the opportunity to talk to the investigator about the conduct of the clinical trial. All my questions were answered satisfactorily. I have understood the purpose of the study and all the details of the procedure.

I had sufficient time to make a decision.

I am aware that I can withdraw my consent to participate in the research at any time and without giving reasons (verbally or in writing) without any disadvantages for me.

I confirm with my signature that I agree to the investigations and to future contacting for long-term research into post-Covid19 syndrome. My participation is free of charge.

I have not been acutely ill within the last 14 days and will inform the research team immediately of any change in my general condition during the examination.

I agree to participate in the above-mentioned study on a voluntary basis.

I have received a copy of the subject information and consent form. One copy remains at the Department

....
Date Signature of the **patient**

I have conducted the informed consent interview and obtained the informed consent form.

....
Date Signature of the **examiner** providing information

Data protection consent for the use of a wearable and mobile phone applications for online support as part of the study "Randomised comparison of telemedicine-supported psychotherapy and exercise therapy and their combination in patients with post-Covid19 syndrome (TELPOCO)"

....

Name of the patient in block capitals

Your data recorded by the wearable will be stored and processed in pseudonymised form (i.e. in a form in which identification features such as name and address are replaced by an identifier - e.g. a code number - so that an assignment to a person is only possible via further aids - such as a reference list - and only within the MHH) on a server outside the MHH in accordance with data protection regulations, in particular the GDPR. Synchronisation takes place via the Fitrockr Hub app using the wearable and a mobile device. The Fitrockr Hub app must be installed on the mobile device. To analyse the training data, your health and activity data is transferred to a Fitrockr server.

Only direct employees of the Clinic for Rehabilitation and Sports Medicine MHH have access to the pseudonymised data. The assignment of the pseudonymised data to your person is also only carried out by employees of the Clinic for Rehabilitation and Sports Medicine.

The recorded and transmitted data includes activity data such as steps and duration of an activity, as well as health data such as your heart rate and the GPS data of your movements. The GPS data of your movement is only recorded if you manually activate a sports activity on the wearable. The GPS recording function can also be deactivated.

I understand that the data that I will disclose as part of the training therapy will be collected and analysed in pseudonymised form. This means in a form in which identification features such as name, date of birth and address are replaced by a code number, so that an assignment to my person is only possible via a further reference list. Only the employees of the Clinic for Rehabilitation and Sports Medicine can verify my identity. In the event of publication of the project results, the confidentiality of personal data will be maintained. The Data Protection Act of the State of Lower Saxony applies.

The use of the information about my health and activity data is carried out in accordance with legal provisions and, in accordance with Article 6 para. 1 lit. a of the GDPR, requires the following voluntary declaration of consent before participation in the clinical study, i.e. without the declaration of consent, participation in the study cannot take place.

I am aware that this consent can be revoked at any time in writing or verbally without giving reasons and without any disadvantages for me. This does not affect the legality of the data processing carried out up to the point of revocation. In this case, I can decide whether the data collected from me should be deleted or may continue to be used in anonymised form for the purposes of the study.

I have been informed and agree that my data will be stored for at least ten years after completion or cancellation of the study in accordance with the law. After this period, my personal data will be deleted, unless there are legal, statutory or contractual retention periods to the contrary.

I have the right to inspect the data collected during the study. Should I discover any errors in the data, I have the right to have these corrected by the study doctor. I have the right to receive information about the personal data in question (including a copy free of charge) and, if necessary, to request its correction or deletion. Furthermore, you have the right to restrict the processing of your personal data (Art. 18 GDPR), to data portability (Art. 20 GDPR) and a general right to object (Art. 21 GDPR).

I am aware that my participation in this study is voluntary and that I can withdraw my consent at any time without giving reasons and without incurring any disadvantages.

I agree to the collection of my personal data as described above.

....

Date Signature of the **patient**
